# Control Region Variability of Haplogroup C1d and the Tempo of the Peopling of the Americas

**DOI:** 10.1371/journal.pone.0020978

**Published:** 2011-06-13

**Authors:** Gonzalo Figueiro, Pedro C. Hidalgo, Mónica Sans

**Affiliations:** Departamento de Antropología Biológica, Facultad de Humanidades y Ciencias de la Educación, Universidad de la República, Montevideo, Uruguay; Institut de Biologia Evolutiva - Universitat Pompeu Fabra, Spain

## Abstract

**Background:**

Among the founding mitochondrial haplogroups involved in the peopling of the Americas, haplogroup C1d has been viewed as problematic because of its phylogeny and because of the estimates of its antiquity, apparently being much younger than other founding haplogroups. Several recent analyses, based on data from the entire mitochondrial genome, have contributed to an advance in the resolution of these problems. The aim of our analysis is to compare the conclusions drawn from the available HVR-I and HVR-II data for haplogroup C1d with the ones based on whole mitochondrial genomes.

**Methodology/Principal Findings:**

HVR-I and HVR-II sequences defined as belonging to haplogroup C1d by standard criteria were gathered from the literature as well as from population studies carried out in Uruguay. Sequence phylogeny was reconstructed using median-joining networks, geographic distribution of lineages was analyzed and the age of the most recent common ancestor estimated using the *ρ*-statistic and two different mutation rates. The putative ancestral forms of the haplogroup were found to be more widespread than the derived lineages, and the lineages defined by np 194 were found to be widely distributed and of equivalent age.

**Conclusions/Significance:**

The analysis of control region sequences is found to still harbor great potential in tracing microevolutionary phenomena, especially those found to have occurred in more recent times. Based on the geographic distributions of the alleles of np 7697 and np 194, both discussed as possible basal mutations of the C1d phylogeny, we suggest that both alleles were part of the variability of the haplogroup at the time of its entrance. Moreover, based on the mutation rates of the different sites stated to be diagnostic, it is possible that the anomalies found when analyzing the haplogroup are due to paraphyly.

## Introduction

The estimation of the antiquity of the human peopling of the Americas has relied heavily on the coalescence ages of the four major founding mitochondrial DNA (mtDNA) haplogroups A, B, C and D [1] (the American variants of which are now named A2, B2, C1, and D1 [2]), with various ages and mechanisms of entrance proposed. Although the most parsimonious mechanism proposed for the initial peopling of the Americas is that of a single migration event [3]–[6], two- [1], [7]–[9] and four-migration [10] models have also been proposed. Moreover, the antiquity of the migration(s) is still subject of much debate. When using mtDNA data, the accuracy of the estimation is based on the resolution of the data and on the assumption that the Time to the Most Recent Common Ancestor (TMRCA) of the haplogroup in question is as close as possible to the time of arrival of its carriers. Haplogroup C1 in particular has recently been consistently subdivided into four haplogroups originated in Beringia: C1a, C1b, C1c, and C1d. The last three are considered as founding American haplogroups, while C1a would have experienced an ancient “backflow” into Asia, not being found in America [11].

Tamm et al. [11] calculated the average coalescence time of the founding Native American C1 haplogroups to be 13,900±2,700 years before present (yBP). Based on analyses of complete mitochondrial genomes, haplogroup C1d was initially defined by a mutation at nucleotide position (np) 7697 in the coding region [11]. Later, an A→G substitution at np 16051, located in the hypervariable region I (HVR-I) was included as a criterion [12]. More recently, this substitution was determined to be at the base of the evolutionary history of the haplogroup, with the 7697A mutation as a later event [13]. Subsequent, more comprehensive analyses of complete C1d mitochondrial genomes defined a singular lineage named C1d1, and a paragroup named C1d* lacking the 7697A mutation [14]. Moreover, the possibility of a control region mutation in np 194 at the root of the haplogroup was discussed, but left open.

As the vertebrate mitochondrial genome is highly mutable, uniparental and non-recombinant, the analysis of whole genomes provides high-resolution data for the analysis of population histories. In spite of these obvious advantages, much of current sequence data refers only to the control region (CR), operationally easier to amplify, sequence, and analyze. Hypervariable regions (HVR) I and II, included in the CR, concentrate approximately 40% of the known variability in 4% of the genome [15]. In this paper, we analyze a set of HVR-I and HVR-II haplogroup C1d sequences, seeking to compare the results with the inferences drawn from whole-genome analyses, and discussing the potential of control region sequences in the tracing of local microevolutionary phenomena.

## Materials and Methods

### Published sequences

**Figure 1:**
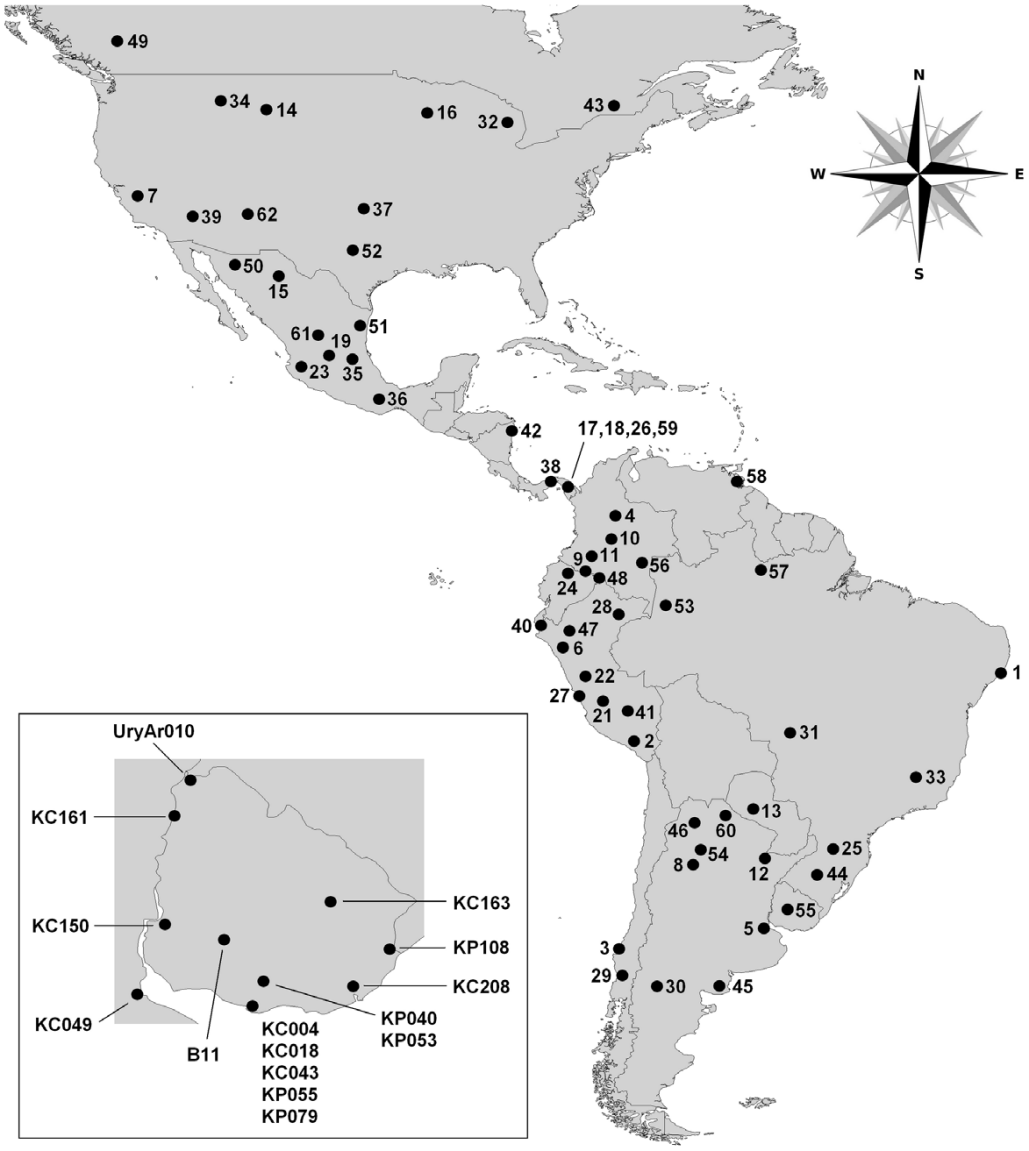
Geographic location of the populations carrying haplogroup C1d. The numbers correspond to those found in Table S1. *Inset:* Birthplaces of the individuals whose sequences were obtained in this study (see Table 1 for details).

North, Central and South American HVR-I (N = 170) and HVR-II (N = 101) sequences belonging to haplogroup C1d as defined by the 16051G mutation plus at least three of the four HVR-I mutations found in haplogroup C1 (16223T, 16298C, 16325C, and 16327T) were collected from the literature (Figure 1; Supporting information, Table S1).

### Unpublished sequences

Sequences (N = 15) selected for this study (Table 1; Figure 1, inset), all belonging to haplogroup C and carrying the 16051G mutation, come from Uruguayan individuals sampled as part of several different population studies conducted during recent years. In all cases, familial data was gathered, and blood samples were collected after obtaining written informed consent for population studies. Total genomic DNA from blood samples was extracted according to the “salting out” method [16]. HVR-I and HVR-II were amplified by PCR and sequenced in separate, overlapping fragments (15996–16498 for HVR-I and 16347–397 for HVR-II). The PCR products, purified using silica columns, were sent to be sequenced in an external service (Macrogen Inc., Seoul, South Korea).

**Table 1:** Sequences obtained in this study.

Sample ID	Birthplace	64	73	194	195	228	249	263	290	291	310i	315i	350	7697	16051	16092	16129	16140	16172	16188	16192	16209	16221	16223	16259	16266	16271	16287	16288	16298	16311	16325	16327
rCRS		C	A	C	T	G	A	A	A	A	-	-	A	G	A	T	G	T	T	C	C	T	C	C	C	C	T	C	T	T	T	T	C
KC004	Montevideo, Uruguay	.	G	T	.	.	d	G	d	d	.	C	.	A	G	.	.	.	.	.	.	.	.	T	.	.	.	T	.	C	C	C	T
KC018	Montevideo, Uruguay	.	G	T	.	.	d	G	d	d	.	C	.	.	G	.	.	C	.	.	.	C	.	T	.	.	.	.	C	C	.	C	T
KC043	Montevideo, Uruguay	.	G	T	C	.	d	G	d	d	C	C	G	A	G	.	.	.	.	.	.	.	.	T	T	.	C	.	.	C	C	C	T
KC049	Buenos Aires, Argentina													A	G	.	.	.	.	.	.	.	.	T	.	.	.	.	.	C	.	C	T
KC150	Mercedes, Uruguay	.	G	T	C	.	d	G	d	d	C	C	.	A	G	.	.	.	.	.	.	.	.	T	.	T	.	.	.	C	.	C	T
KC161	Constitución, Uruguay	T	G	T	.	.	d	G	d	d	.	C	.	A	G	.	.	.	.	.	.	.	.	T	.	.	.	.	.	C	.	C	T
KC163	Tupambaé, Uruguay	T	G	T	C	.	d	G	d	d	C	C	.	A	G	.	.	.	.	.	.	.	.	T	.	.	.	.	.	C	.	C	T
KC208	Rocha, Uruguay	.	G	T	.	.	d	G	d	d	.	C	.	.	G	.	.	C	.	.	.	.	.	T	.	.	.	.	C	C	.	C	T
KP040	Canelones, Uruguay													A	G	.	.	.	.	.	T	.	T	T	.	.	.	.	.	C	.	C	T
KP053	Canelones, Uruguay													A	G	.	.	.	.	T	.	.	.	T	.	.	.	.	.	C	.	C	T
KP055	Montevideo, Uruguay													A	G	.	A	.	.	.	.	.	.	T	.	.	.	.	.	C	.	C	T
KP079	Montevideo, Uruguay													A	G	.	.	.	.	.	.	.	.	T	.	.	.	.	.	C	.	C	T
KP108	Chuy, Uruguay													A	G	.	.	.	C	.	.	.	.	T	.	.	.	.	.	C	.	C	T
B11	Trinidad, Uruguay		G	T	.	.	d	G	d	d	.	C	.	.	G	.	.	C	.	.	.	.	.	T	.	.	.	.	C	C	.	C	T
UryAr010	Artigas, Uruguay		G	T	C	A	d	G	d	d	.	C	.		G	C	.	.	.	.	.	.	.	T	.	.	.	.	.	C	.	C	T

Dots indicate identity with the revised Cambridge Reference Sequence (rCRS). Blank spaces indicate lack of data.

In addition, the samples were tested for the presence of diagnostic haplogroup C 13262 *Hinc*II(-) polymorphism and for the 7697 G→A mutation. The latter was detected by digesting a 184 bp PCR segment carrying the polymorphic site (amplified using primers 7630F: 5′-CAAGACGCTACTTCCCC-3′ and 7778R: 5′-GGCGGGCAGGATAGTTC-3′ specifically designed for this study) overnight with 1 unit of *Mae*I restriction enzyme. The presence of the mutation (7694 *Mae*I(-)) was verified by agarose gel electrophoresis of the digested PCR products.

### Analysis of the set of sequences

#### HVR-I analysis

The sequences were truncated to 315 bp, spanning from np 16051 to np 16365 (according to the Revised Cambridge Reference Sequence [17]). Using the trimmed set of sequences, a median-joining network [18] was generated using Network 4.5 (http://www.fluxus-engineering.com/sharenet.htm). The network was further processed using the maximum parsimony (MP) calculation [19] and visualizing the minimum number of trees necessary for explaining the original network. Weights were assigned to the sites in an inverse proportion to the relative mutation rates given by Meyer et al. [20] (i.e., if a given site had the average rate, a weight of 8 was assigned, if the rate was twice the average the assigned weight was 6, and so on).

Although the exclusion of data is never desirable, the high mutability of the control region makes the proper evaluation of multiple hits difficult. Therefore, seeking a compromise between avoiding multiple hits and including as much informative data as possible, a careful selection of sites to be excluded was made, based on previous analyses which provide a site-by-site evaluation of mutational hotspots in the control region. Sites 16093, 16129, 16189, 16311, and 16362 (five sites out of a total 50 variable positions in the database) were purposefully ignored, as they appear mutated on average 88 times (4%) in the data set analyzed by Soares et al. [15] and are classified as fast-evolving in at least one of three previous studies [20]–[22].

#### HVR-I and HVR-II joint analysis

For those samples with data for both HVR-I and HVR-II, HVR-I was defined as spanning np 16024 to np 16383, and HVR-II from np 57 to np 372 [11]. A median-joining network was generated as described above, and in addition to the HVR-I sites mentioned, indels in polynucleotide stretches and HVR-II sites 146, 150, 152, and 195 (nine sites in a total 62 variable positions) were ignored for the construction of the network. These sites appear mutated 107 times (5%) [15], and have an estimated mutation rate six times above average in a previous study [20].

#### Geographic distribution

The geographic coordinates of the populations listed in Table S1 were registered according to the exact place of the listed locality or, in the case of broader regions (i.e. administrative divisions or even countries), its approximate geographic centre. These coordinates were used for plotting the location of specific variants, as well as for calculating mean linear distances between populations carrying the same variant. Calculation of linear distances based on geographic coordinates and statistical analysis (Mann-Whitney U test) of the distances were carried out using the *gmt* and *base* packages in the R 2.8.1 environment (http://www.r-project.org).

#### TMRCA estimates

Based on the networks, possible ancestral lineages were identified and the diversification time of these lineages were calculated using the *ρ*-statistic [5], [23] and two mutation rates. The first rate, of 11.7 mutations/base/million generations [24], is based on genealogical inference, and an average pre-industrial generation span of 25 years [25] was assumed. For this rate, calculations were made using the raw sequences. The other study is based on phylogenetic inference, and estimates an average rate of 9.883×10^−2^ mutations/base/million years [15]. In this case, for calculating *ρ*, indels in general and a number of sites, estimated to have mutation rates three times or more above the average of their respective region [20], were left out of the calculation: these sites are 16093, 16126, 16129, 16148, 16163, 16172, 16183, 16187, 16189, 16192, 16223, 16230, 16270, 16278, 16293, 16294, 16309, 16311, 16319, and 16362 (HVR-I) and 64, 73, 93, 146, 150, 151, 152, 153, 182, 185, 186, 189, 195, 198, 199, 200, 207, 236, 247, 263, and 316 (HVR-II). Standard error for the TMRCA estimate was based on the variance estimate of *ρ* [26]. All calculations were carried out using the *ape* 2.2–4 [27] and *base* packages in the R 2.8.1 environment.

## Results

### HVR-I analysis

**Figure 2:**
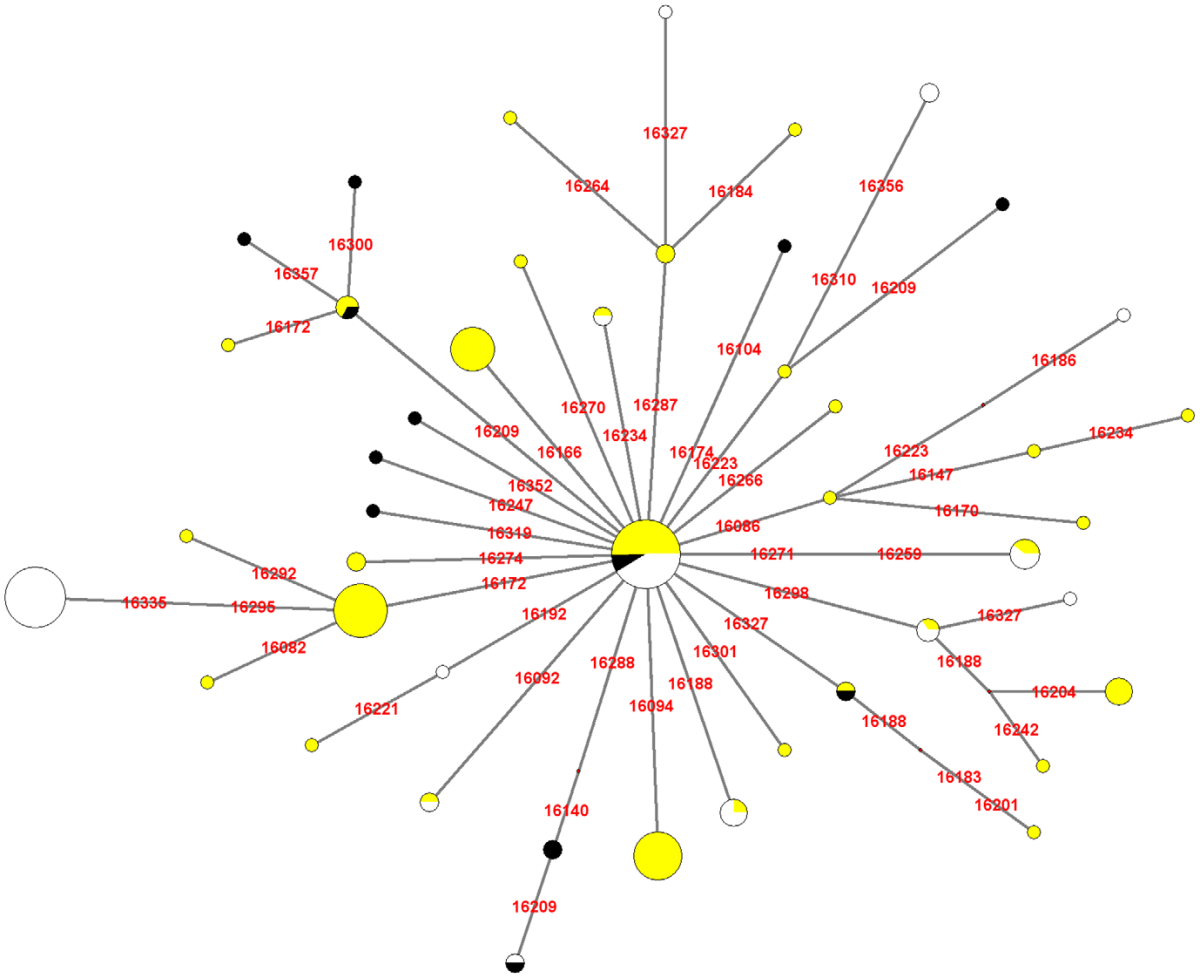
Median-Joining Network generated from the compiled HVR-I sequences. The central lineage carries the mutations 16051G, 16223T, 16298C, 16325C, and 16327T. Yellow: fraction of the lineage carrying the 7697G→A mutation. Black: fraction of the lineage carrying the ancestral 7697G allele. White: fraction of the lineage lacking data on the state of np 7697.

The Median-Joining Network generated using the HVR-I sequences was comprised by a minimum of 5 MP trees, one of which is shown in Figure 2. Some of the sites providing multiple links (16209, 16223, 16298 and 16327) are documented as very mutable [15], [20]–[22], so they could be witnessing recurrent mutation. Nonetheless, they affect secondary branches: the primary branches (i.e., leading to the root) are highly consistent. Moreover, the exclusion of the five hypervariable sites 16093, 16129, 16189, 16311, and 16362 had the effect of drastically reducing the total number of back mutations necessary to explain the relationships between haplotypes, from 40 to 16 (data not shown).

### HVR-I and HVR-II analysis

**Figure 3:**
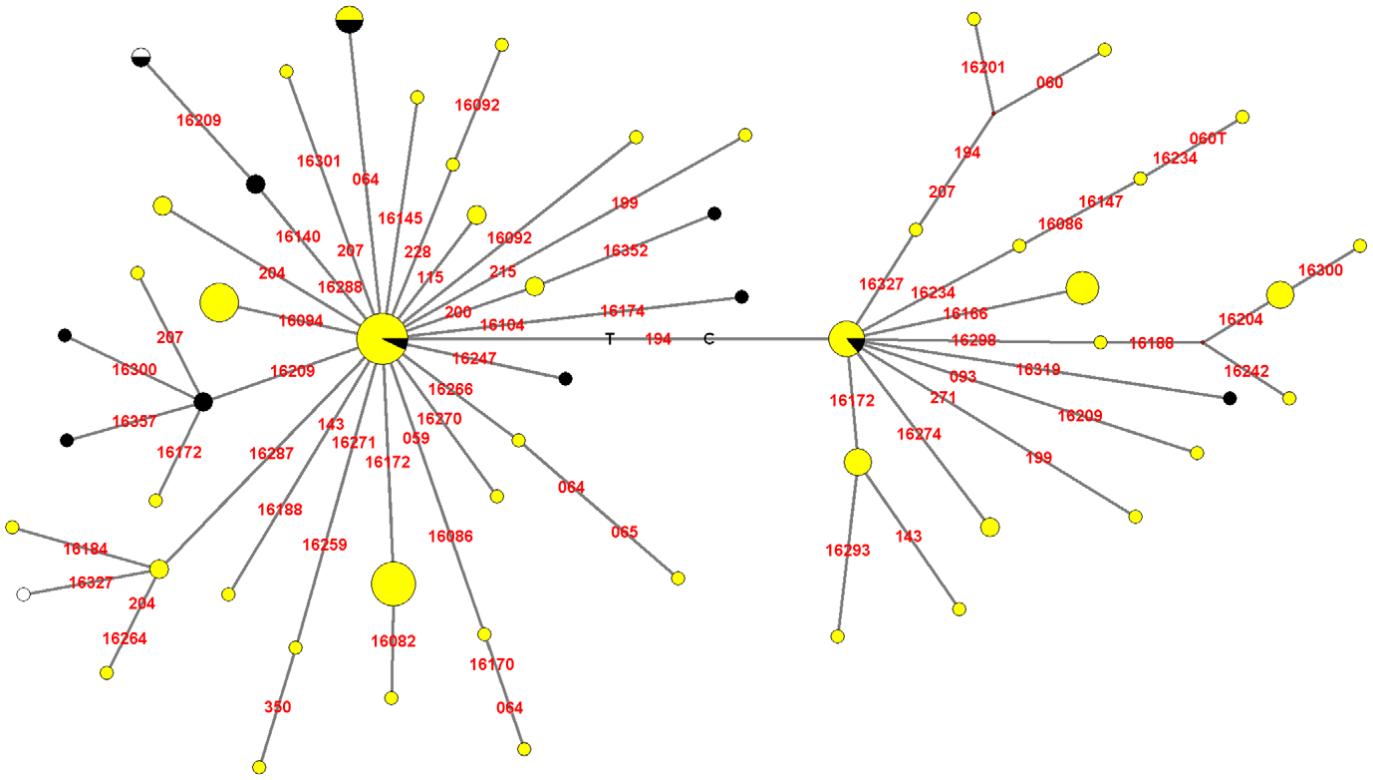
Median-Joining Network generated from the compiled HVR-I and HVR-II sequences. Yellow: fraction of the lineage carrying the 7697G→A mutation. Black: fraction of the lineage carrying the ancestral 7697G allele. White: fraction of the lineage lacking data on the state of np 7697.

The Median-Joining Network generated using the HVR-I and HVR-II sequences employing the whole sample was comprised by a minimum of 4 MP trees, one of which is shown in Figure 3. The network shows quite clearly the presence of two ancestral lineages, one characterized by a cytosine in np 194 and the other characterized by a thymine in this position. This mutation has been considered to be a possible basal state for the C1d clade [14]. The exclusion of the five hypervariable HVR-I sites plus the additional four HVR-II sites 146, 150, 152, and 195 reduced the number of minimum back mutations from 46 to 20 (data not shown).

### Geographic distribution

**Figure 4:**
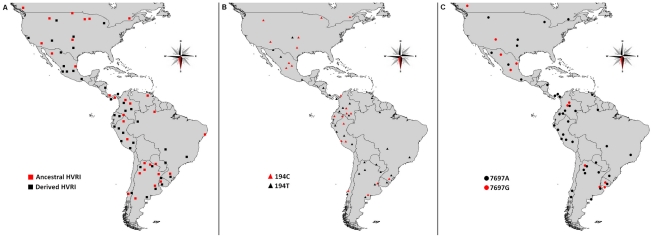
Distribution of C1d lineages in the Americas. A: Distribution of the ancestral and derived lineages as defined using HVR-I only. B: Distribution of lineages carrying the 194C and 194T alleles. C: Distribution of lineages with and without the 7697G→A mutation.

The distribution of the ancestral (16051G-16223T-16298C-16325C-16327T) and derived lineages for the HVR-I analysis is shown in Figure 4A. Both types of lineages can be found throughout the Americas. The ancestral lineage has a large geographic distribution, with a mean interpopulation distance of 4,762 Km and 10% of the distances below 1,000 Km. In contrast to the ancestral lineage, populations sharing a same derived lineage are located on average 2,786 Km away, with 28% of the distances being less than 1,000 Km. The difference between the distance distributions is statistically significant (U = 14,027, Z = 5.63, p<.0001).

For the HVR-I+HVR-II analysis, the geographic location of the ancestral lineages carrying 194C and 194T are shown in Figure 4B; they are widely distributed throughout the continent without any seeming differential pattern. As for the difference between the two ancestral (73G, 194C/T, 249d, 263G, 290–291d) and derived lineages, once again the ancestral lineages show a greater geographic distribution. For the 194C lineages, the ancestral node shows a mean interpopulation distance of 4,511 Km, while the derived lineages have a mean interpopulation distance of 2,077 Km. The difference between the distance distributions is statistically significant (U = 24, Z = 2.34, p = 0.0193). The same applies to the 194T lineages (mean distance – ancestral lineage: 3,138 Km; mean distance – derived lineages: 1,694 Km; U = 375, Z = 2.17, p = 0.03).

### TMRCA estimates

The *ρ* estimates and the calculated times to the most recent common ancestor of root lineages for the HVR-I and HVR-I+HVR-II phylogenies are shown in Table 2. Based on the phylogenetic and genealogical mutation rate estimates, four TMRCA were calculated: The first, based on the HVR-I analysis, estimates the divergence time from the ancestral 16051G lineage. The other three are based on the HVR-I+HVR-II analysis, the first assuming 194T as the root lineage and the 194C lineage as the result of a back mutation, and the other two considering 194C and 194T separately.

**Table 2:** *ρ*-statistics and TMRCA estimates for various root lineages.

Lineage	ρ*	σ*	TMRCA*	ΔT*	ρ**	σ**	TMRCA**	ΔT**
HVRI	1.484	0.513	10063.2	3481.6	0.863	0.425	16904.5	8332.6
HVRI+II	2.257	0.643	7717.7	2198.4	1.406	0.582	22616.6	9365.9
HVRI+II 194C	2.086	0.680	7130.6	2325.9	1.086	0.503	18811.1	8715.9
HVRI+II 194T	1.895	0.663	6436.5	2251.8	1.171	0.545	20289.7	9446.5

*TMRCA based on a genealogical mutation rate [24]; fast-evolving sites included.

**TMRCA based on a phylogenetic mutation rate [15]; fast-evolving sites not included.

## Discussion

### Variability and distribution of haplogroup C1d

The geographic distributions of the HVR-I 16051G motif (Figure 4A) on one hand, and of the 194T and 194C lineages (Figure 4B) on the other are wide enough to make it reasonable to assume that haplogroup C1d entered the continent as a part of the initial peopling of the Americas. It can also be inferred that the haplogroup had already accumulated some amount of variability prior to the entrance to the continent, which implies that the TMRCA of C1d is not equivalent to its age of arrival. More research will have to be done in order to establish the amount of “maturation” of Beringian haplogroups prior to the peopling [11], [28]. Its extent would be heavily dependent on the time of the purported standstill (most probably a minimum of 5,000 years [29]) and on the effective size of the group that entered the continent, which to this day is a subject of debate, ranging from a magnitude of a hundred individuals [30]–[32] to 5–10,000 individuals [4], [28]. We wish to stress that given the *maximum* registered size of 180 individuals in modern hunter-gatherer groups during the most aggregated phases of their mobility cycle [33], [34], effective population sizes beyond the hundreds for the human groups peopling the Americas are far from likely. Nonetheless, if the peopling of the continent occurred as a more or less continuous flow during the period of time in which passage from Asia was possible, the effective size of the resulting metapopulation would be higher.

### Time estimates, molecular clock and the tempo of the peopling of the Americas

The age estimates using the two mutation rates (Table 2) vary almost twofold, even when the genealogical mutation rate was applied including hypervariable sites, i.e., on raw sequences. This leads us to two important considerations. A recent study [35] shows that there is a sharp increase in mtDNA mutation rate for human populations younger than 15,000 years, attributed to a series of bottlenecks up to 50,000 yBP. The archaeological estimates of human settlement in South America at 15,000 yBP imply that the entrance into North America must be older, but the question of how much older is subject to debate. Considering point estimates of 12,900–15,000 yBP for the date of the entrance of man to the continent [35], American populations would be located chronologically at the exact point where pedigree rates are useful. Therefore, we must bear in mind that the ages calculated on pedigree-based rates might be underestimations. It must nonetheless be noted that a HVR-I mutation rate calibrated using the sequence of an Early Holocene specimen [36] yields an age estimate equivalent to the pedigree-based rate used in this work with the generation interval raised to 30 years (data not shown). Moreover, regarding recent observations by Cox [37] on the Type I error associated to molecular datings based on the *ρ*-statistic, none of the age estimates can be taken into account with certainty, and alternatively, more computer-intensive estimates should be employed.

However, some inferences are possible based on present data. As for the potential of the hypervariable region as a “proxy” for mitochondrial variability as a whole, it is obvious that the standard error of variability estimates based on a mere 4% of the genome will be much higher than the standard error of whole-genome estimates. Also, the phylogenetic resolution of analyses based on whole genomes will certainly be higher: only 49 of the 70 lineages defined in the detailed tree in the analysis made by Perego et al. [14] are defined by HVR-I mutations, HVR-II mutations, or both. Moreover, whole-genome sequences tell a different story than control region sequences, given that the former can carry evidence of selection, which cannot be seen in the latter. Nevertheless, if we compare the ages inferred from the HVR sequences with the ones inferred from whole-genome studies using the same rate [15], there is a difference of 1,400 years, which comfortably falls within the most stringent standard error [14]. Moreover, it must be noted that of the 49 lineages mentioned above, 42 were found to be reproduced in our HVR-I+HVR-II network (Figure 3). The implication of this is that, for studies regarding the peopling of the continent as a whole, control region sequences still have a lot to offer as a relatively fast and easy way to explore the mitochondrial variability within a given region.

As for the tempo of the peopling of the Americas, it has recently been explicitly referred to as “swift” without further detail [11]. Based on the near identity of the age of the MRCA of the South American C1d lineages (Table 3) and the estimate for the American phylogeny (Table 2), we propose that the measure of “swift” could be on the order of magnitude of a millennium, as was initially postulated for the Clovis expansion [38] – although we are not stating that the Clovis expansion and the initial peopling of the Americas were simultaneous events. Furthermore, present genetic variability in America can be explained by coastal migration, which would also have the outcome of a relatively fast rate of advance, as has been shown by simulations [39].

**Table 3:** *ρ*-statistics and TMRCA estimates for various root lineages, using lineages present only in South America.

Lineage	ρ*	σ*	TMRCA*	ΔT*	ρ**	Σ**	TMRCA**	ΔT**
HVRI	1.528	0.570	10362.0	3864.9	0.850	0.465	16664.6	9102.5
HVRI+II	2.068	0.644	7068.6	2201.7	1.243	0.582	19999.4	9370.3
HVRI+II 194C	1.952	0.869	6674.8	2970.8	0.905	0.593	15675.9	10271.8
HVRI+II 194T	1.825	0.657	6201.0	2230.4	1.190	0.541	20626.2	9370.7

*TMRCA based on a genealogical mutation rate [24]; fast-evolving sites included.

**TMRCA based on a phylogenetic mutation rate [15]; fast-evolving sites not included.

Regarding certain details on the phylogeny of haplogroup C1d, the HVR-I+HVR-II network shown in Figure 3, as well as the related *ρ*-statistics (Tables 2 and 3) show that the average evolution of the 194C lineages is only slightly lower than the one in 194T lineages – and higher if hypermutable sites are taken into account – implying that C1d most probably retained a polymorphism in np 194, with 194C as the ancestral state. Frequent back mutations in 194 would result in a substantially lower amount of mutations in 194C lineages. The excess of mutations in 194C lineages is apparent when *ρ* is calculated on pooled data using 194T as the root, which pushes the TMRCA back to 22,000 years. Interestingly, the use of 194C as the root lineage on pooled data (not shown) does only increase the *ρ* value, which implies that we are observing a major, and rather non-parsimonious, ancestral polymorphism in haplogroup C1d. Further still, although the confidence intervals for the ages of the MRCA of the 194T and 194C variants overlap, the mean difference might be indicating that the 194C and 194T C1d variants might have had independent origins. This inference about the status of the 194T mutation in the phylogeny of C1d is a major difference with respect to conclusions reached by previous work [14]. It could be a difference due to the different methodological approaches employed in the construction of the phylogenies, and to the different processes that can be observed by analyzing a non-coding sequence compared to non-coding and coding sequences together.

### On the status of 7697G and 16051 in the C1d clade

The distribution of the 7697A/G polymorphism, which was not considered in our analyses, is rather irregular in the C1d phylogenies (shown as black portions of the lineages in Figures 2 and 3), and has a pan-American distribution (Figure 4C) as noted in previous work [14]. Taking the three mutations considered at different times as being ancestral to haplogroup C1d (16051G [12], [13], 194T [14] and 7697A [11]), it must be noted that 7697A→G is a mutation which leads to an amino acid change, while the other two are non-coding mutations. Of the latter, 194 is a site with an average mutation rate for HVR-II [20] and was observed mutated 12 times (0.55%) in a total 2,196 mtDNA genomes [15], and 16051 has a mutation rate four times above average for HVR-I [20] (that is, double of that of HVR-II [15]) and was observed mutated 21 times in 2,196 sequences (0.96%) [15]. This taken into account, back mutations in np 16051 are the most likely. Thus, we must consider the possibility of C1d being a paraphyletic group, and its variability being under-represented.

### On the potential of localized subhaplogroups

As a final note, although control region sequences may lack the power of whole-genome analyses in tracing discrete migration events in the earlier stages of the peopling of the Americas [40], they still have a great potential in the investigation of more recent microevolutionary phenomena. In this case, we want to point out two geographically restricted lineages which might be of great interest: One is defined by the HVR-II 194T-195C motif, which is found in Argentina (especially the Northwestern part of the country) and Uruguay. The other is defined by the HVR-I 16140C-16288C motif, which has hitherto been found only in Uruguayan samples. Sequences as these might serve as “private” variants useful for tracing population and individual movements, even in historic times.

### Accession numbers

The Uruguayan sequences reported in this paper have been submitted to GenBank, with accession numbers HQ848717-HQ848739 and JF317298.

## Supporting Information

Table S1 Ethnic groups and regions in which haplogroup C1d has been found.
(DOC)
