# Supplementary material for: Control Region Variability of Haplogroup C1d and the Tempo of the Peopling of the Americas
Source: PLoS One. 2011 Jun 13;6(6):e20978. doi: 10.1371/journal.pone.0020978 (PMC3113844; doi:10.1371/journal.pone.0020978)
Supplement: Table S1 — Ethnic groups and regions in which haplogroup C1d has been found. (DOC) [file pone.0020978.s001.doc]

|  | **Group or region** | **Segment** | **N** | **Reference** |
| --- | --- | --- | --- | --- |
| 1 | Alagoas (NE Brazil) | HVR-I | 1 | [1] |
| 2 | Arequipa/Tayacaja (S Peru) | HVR-I | 1 | [2] |
| 3 | Biobío (Chile) | HVR-I and HVR-II | 1 | [3] |
| 4 | Boyaca (Colombia) | HVR-I and HVR-II | 1 | [3] |
| 5 | Buenos Aires (Argentina) | HVR-I and HVR-II | 6 | [3] |
| 6 | Cajamarca (Peru) | HVR-I and HVR-II | 1 | [3] |
| 7 | California (United States) | HVR-I | 1 | [4] |
| 8 | Catamarca (NW Argentina) | HVR-I and HVR-II | 4 | [3,5] |
| 9 | Cofan (Ecuador) | HVR-I and HVR-II | 2 | [5] |
| 10 | Colombia | HVR-I and HVR-II (7) | 9 | Unpublished(*); [3] |
| 11 | Coreguaje (Colombia) | HVR-I and HVR-II | 11 | [5] |
| 12 | Corrientes (Argentina) | HVR-I and HVR-II | 2 | [3] |
| 13 | Chaco (Paraguay) | HVR-I and HVR-II | 1 | [3] |
| 14 | Cheyenne (NE United States) | HVR-I | 1 | [6] |
| 15 | Chihuahua (Mexico) | HVR-I and HVR-II | 1 | [3] |
| 16 | Chippewa (NE United States) | HVR-I | 15 | [6] |
| 17 | Darien (Panama) | HVR-I and HVR-II | 1 | [3] |
| 18 | Embera (Panama / Colombia) | HVR-I and HVR-II (4) | 8 | [5,7] |
| 19 | Guanajuato (Mexico) | HVR-I and HVR-II | 1 | [3] |
| 20 | Hispanic (United States) | HVR-I and HVR-II (3) | 4 | [8,9] |
| 21 | Huancavelica (Peru) | HVR-I and HVR-II | 1 | [3] |
| 22 | Huanucu (Peru) | HVR-I and HVR-II | 1 | [3] |
| 23 | Huichol (W Mexico) | HVR-I | 3 | [10] |
| 24 | Imbabura (Ecuador) | HVR-I and HVR-II | 1 | [3] |
| 25 | Kaingang (S Brazil) | HVR-I | 22 | [11] |
| 26 | Kuna Yala (Panama) | HVR-I and HVR-II | 1 | [3] |
| 27 | Lima (Peru) | HVR-I and HVR-II | 1 | [3] |
| 28 | Loreto (Peru) | HVR-I and HVR-II | 2 | [3] |
| 29 | Los Lagos (Chile) | HVR-I and HVR-II | 2 | [3] |
| 30 | Mapuche (S Argentina) | HVR-I | 1 | [3] |
| 31 | Mato Grosso do Sul (Brazil) | HVR-I and HVR-II | 1 | [3] |
| 32 | Michigan (United States) | HVR-I and HVR-II | 1 | [3] |
| 33 | Minas Gerais (Brazil) | HVR-I and HVR-II | 1 | [3] |
| 34 | Montana (United States) | HVR-I and HVR-II | 1 | [3] |
| 35 | Nahua (E Mexico) | HVR-I | 1 | [10] |
| 36 | Oaxaca (Mexico) | HVR-I and HVR-II | 1 | [3] |
| 37 | Oklahoma (United States) | HVR-I and HVR-II | 2 | [3] |
| 38 | Panama | HVR-I and HVR-II | 1 | [3] |
| 39 | Pima (SW United States) | HVR-I | 1 | [10] |
| 40 | Piura (Peru) | HVR-I and HVR-II | 1 | [3] |
| 41 | Puca Puca (Peru) | HVR-I and HVR-II | 1 | [3] |
| 42 | Puerto Cabezas (Nicaragua) | HVR-I and HVR-II | 1 | [3] |
| 43 | Quebec (Canada) | HVR-I and HVR-II | 1 | [3] |
| 44 | Rio Grande do Sul (Brazil) | HVR-I and HVR-II | 2 | [3] |
| 45 | Rìo Negro (Argentina) | HVR-I and HVR-II | 2 | [3] |
| 46 | Salta (Argentina) | HVR-I and HVR-II | 6 | [3] |
| 47 | San Martín de Pangoa (Peru) | HVR-I | 1 | [2] |
| 48 | Secoya (Ecuador) | HVR-I and HVR-II | 1 | [5] |
| 49 | Shuswap (British Columbia, Canada) | HVR-I | 1 | [12] |
| 50 | Sonora (Mexico) | HVR-I and HVR-II | 1 | [3] |
| 51 | Tamaulipas (Mexico) | HVR-I and HVR-II | 1 | [3] |
| 52 | Texas (United States) | HVR-I and HVR-II | 2 | [3] |
| 53 | Ticuna (Amazonia) | HVR-I | 1 | [13] |
| 54 | Tucumán (NW Argentina) | HVR-I and HVR-II | 1 | [5] |
| 55 | Uruguay | HVR-I and HVR-II | 4 | [3,14] |
| 56 | Vaupes (Colombia) | HVR-I and HVR-II | 2 | [5] |
| 57 | Waiwai (N Brazil) | HVR-I and HVR-II | 1 | [15] |
| 58 | Warao (NE Venezuela) | HVR-I | 2 | [16] |
| 59 | Waunana (Panamá / Colombia) | HVR-I and HVR-II (8) | 13 | [5,7] |
| 60 | Wichi (N Argentina) | HVR-I | 6 | [17] |
| 61 | Zacatecas (Mexico) | HVR-I and HVR-II | 1 | [3] |
| 62 | Zuni (SW United States) | HVR-I and HVR-II | 1 | [5] |

(*)GenBank ID EU566190 and EU566193

**References**

1. Barbosa AB, da Silva LA, Azevedo DA, Balbino VQ, Mauricio-da-Silva L (2008) Mitochondrial DNA control region polymorphism in the population of Alagoas state, north-eastern Brazil. J Forensic Sci 53: 142-146.

2. Fuselli S, Tarazona-Santos E, Dupanloup I, Soto A, Luiselli D, et al. (2003) Mitochondrial DNA Diversity in South America and the Genetic History of Andean Highlanders. Mol Biol Evol 20: 1682-1691.

3. Perego UA, Angerhofer N, Pala M, Olivieri A, Lancioni H, et al. (2010) The initial peopling of the Americas: A growing number of founding mitochondrial genomes from Beringia. Genome Res 20: 1174-1179.

4. Johnson JR, Lorenz JG (2006) Genetics, linguistics, and prehistoric migrations: an analysis of California Indian mitochondrial DNA lineages. J Calif Great Basin Anthropol 26: 33-34.

5. Tamm E, Kivisild T, Reidla M, Metspalu M, Smith DG, et al. (2007) Beringian Standstill and Spread of Native American Founders. PLoS ONE 2: e829.

6. Shook BAS, Smith DG (2008) Using Ancient mtDNA to Reconstruct the Population History of Northeastern North America. Am J Phys Anthropol 137: 14-29.

7. Kolman CJ, Bermingham E (1997) Mitochondrial and Nuclear DNA Diversity in the Choco and Chibcha Amerinds of Panama. Genetics 147: 1289-1302.

8. Parsons TJ (2006) Mitochondrial DNA Genome Sequencing and SNP Assay Development for Increased Power of Discrimination. U.S. Department of Justice. 119 p.

9. Saunier JL, Irwin JA, Just RS, O'Callaghan J, Parsons T, J (2008) Mitochondrial control region sequences from a U.S. 'Hispanic' population sample. Forensic Sci Int Genet 2: e19-23.

10. Kemp BM (2006) Mesoamerica and Southwest prehistory, and the entrance of humans into the Americas: mitochondrial DNA evidence [PhD Dissertation]. Davis: University of California.

11. Marrero AR, Silva-Junior WA, Bravi CM, Hutz MH, Petzl-Erler ML, et al. (2007) Demographic and Evolutionary Trajectories of the Guarani and Kaingang Natives of Brazil. Am J Phys Anthropol 132: 301-310.

12. Malhi RS, Cybulski JS, Tito RY, Johnson J, Harry H, et al. (2010) Brief communication: mitochondrial haplotype C4c confirmed as a founding genome in the Americas. Am J Phys Anthropol 141: 494-497.

13. Torroni A, Schurr TG, Cabell MF, Brown MD, Neel JV, et al. (1993) Asian affinities and continental radiation of the four founding Native American mtDNAs. Am J Hum Genet 53: 563-590.

14. Pagano S, Sans M, Pimenoff V, Cantera AM, Alvarez JC, et al. (2005) Assessment of HV1 and HV2 mtDNA variation for forensic purposes in an Uruguayan population sample. J Forensic Sci 50: 1239-1242.

15. Fagundes NJR, Kanitz R, Eckert R, Valls ACS, Bogo MR, et al. (2008) Mitochondrial Population Genomics Supports a Single Pre-Clovis Origin with a Coastal Route for the Peopling of the Americas. Am J Hum Genet 82: 583-592.

16. Ingman M, Kaessmann H, Pääbo S, Gyllensten U (2000) Mitochondrial genome variation and the origin of modern humans. Nature 408: 708-713.

17. Cabana GS, Merriwether DA, Hunley K, Demarchi DA (2006) Is the genetic structure of Gran Chaco populations unique? Interregional perspectives on native South American mitochondrial DNA variation. Am J Phys Anthropol 131: 108-119.
